# Development and validation of a comprehensive radiomics nomogram for prognostic prediction of primary hepatic sarcomatoid carcinoma after surgical resection

**DOI:** 10.7150/ijms.53602

**Published:** 2021-02-06

**Authors:** Youyin Tang, Tao Zhang, Yunuo Zhao, Zheyu Chen, Xuelei Ma

**Affiliations:** 1 Department of Liver Surgery, Liver Transplantation Center, West China Hospital of Sichuan University, No. 37 GuoXue Alley, Chengdu 610041, China.; 2 West China School of Medicine, West China Hospital, Sichuan University, No. 37 GuoXue Alley, Chengdu 610041, China.; 3 Department of Biotherapy, West China Hospital and State Key Laboratory of Biotherapy, Sichuan University, No. 37 GuoXue Alley, Chengdu 610041, China.

**Keywords:** primary hepatic sarcomatoid carcinoma, prognosis, nomogram, radiomics, clinical decision-making

## Abstract

**Objective:** This study aimed to establish and validate a radiomics nomogram comprised of clinical factors and radiomics signatures to predict prognosis of primary hepatic sarcomatoid carcinoma (PHSC) patients after surgical resection.

**Methods:** In this retrospective study, 79 patients with pathological confirmation of PHSC and underwent surgical resection were recruited. A radiomics nomogram was developed by radiomics signatures and independent clinical risk factors selecting from multivariate Cox regression. All patients were stratified as high risk and low risk by nomogram. Model performance and clinical usefulness were assessed by C-index, calibration curve, decision curve analysis (DCA) and survival curve.

**Results:** A total of 79 PHSC were included with 1-year and 3-year overall survival rates of 63.3% and 35.4%, respectively. The least absolute shrinkage and selection operator (LASSO) method selected 3 features. Multivariate Cox analysis found six independent prognostic factors. The radiomics nomogram showed a significant prediction value with overall survival (HR: 7.111, 95%CI: 3.933-12.858, P<0.001). C-index of nomogram was 0.855 and 0.829 in training and validation set, respectively. Decision curve analysis validated the clinical utility of this nomogram. There was a significant difference in the 1-year and 3-year survival rates of stratified high-risk and low-risk patients in the whole cohort (30.6% vs. 90.1% and 5.6% vs. 62.4%, respectively, P < 0.001).

**Conclusion:** This radiomics nomogram serve as a potential tool for predicting prognosis of PHSC after surgical resection, and help to identify high risk patients who may obtain feeble survival benefit from surgical resection.

## Introduction

Primary hepatic sarcomatoid carcinoma (PHSC) is a rare and complicated type of liver malignant tumor, and the reported incidence was 0.18%-0.7% in all liver malignancy 1, 2. Previous studies found that PHSC is a hybrid-type neoplasm comprised of carcinoma and sarcomatoid components, and can be mainly divided into sarcomatoid hepatocellular carcinoma (SHC), intrahepatic sarcomatoid cholangiocarcinoma (ISCC) and undifferentiated carcinoma by different carcinoma components according to World Health Organization (WHO) classification of digestive tumours 3-5. In addition, the sarcomatoid components of PHSC were different from the conventional sarcomatoid carcinoma, such as osteosarcoma, hepatic follicular dendritic cell sarcoma and other sarcoma, and were usually defined as the composing of spindle cells and ultrastructurally, immunohistochemically and morphologically recognizable epithelial components 6, 7. In the literature review, only few series or case reports of PHSC were reported due to its rarity, and a majority of them studied the special histological or image characteristics of PHSC 5, 8-13, whereas the others discussed the clinical factors relating to its poor prognosis, with the estimating 1-year, 3-year and 5-year overall survival rate of 40%-50%, 17%-21.4% and 14.3%-17% after surgery, respectively 1, 2. The prognosis of PHSC was extremely poor regarding to its histological nature of sarcomatoid carcinoma 8, however, as for the risk factors of survival, these previous prognostic studies were inconclusive because they only pay attention to the clinical factors but ignored the impact of cellular heterogeneity of PHSC on prognosis.

Radiomics is defined as quantitative depicting, which can be interpreted as extraction, interpretation and analyzing of quantitative features of digital medical image to predict clinical outcomes 14. In a sense, radiomics can analyze the heterogeneity of tumor from the image itself 15-17. In recent years, this non-invasive and quantitative evaluation method of tumor heterogeneity has also been developing rapidly, and has been applied in various imaging technologies 18-20. Radiomics can be considered as a prognostic tool with outstanding potential. And Nomogram used clinical indicators or biological attributes through multiple factors regression analysis as well as used number marked line segments to predict the probability of certain events or clinical outcomes based on the values of multiple factors 21. It converted the obscure regression equation into an intuitive and simple graph, which made the results of the prediction model easier to understand 22. Previous study found that a radiomics nomogram comprising of clinical factors and radiomics signature can provide higher prediction value than either clinical model or radiomics model in hepatocellular carcinoma and intrahepatic cholangiocarcinoma 23-25. However, to our knowledge, there was no radiomics nomogram regarding to the survival prediction of PHSC after surgical resection.

The goal of this study was to develop a nomogram based on enhanced computed tomographic (CT) radiomics and clinical factors to predict prognosis of patients with PHSC after surgical resection, identifying high risk patients who may obtain feeble survival benefit from surgical resection.

## Materials and Methods

### Study design and patient selection

This study was approved by the Ethics Committee of West China Hospital, Sichuan University. No inform consent required because of no individual information was disclosed. In this study, we retrospectively reviewed all patients with pathological proven of primary hepatic sarcomatoid carcinoma (PHSC) according to WHO classification 3, 4 and received curative-intent surgery without preoperative radiochemotherapy during January 2010 to December 2017 in West China Hospital of Sichuan University. The pathological slices of PHSC could found sarcomatoid components and liver carcinoma components. The sarcomatoid components of PHSC were composing of spindle cells and ultrastructurally, immunohistochemically and morphologically recognizable epithelial components. And other biomarkers, such as cytokeratin 7, cytokeratin 19, cytokeratin 20, epithelial membrane antigen, vimentin, HepPar1 and smooth muscle actin might be positive.

**Figure 1:**
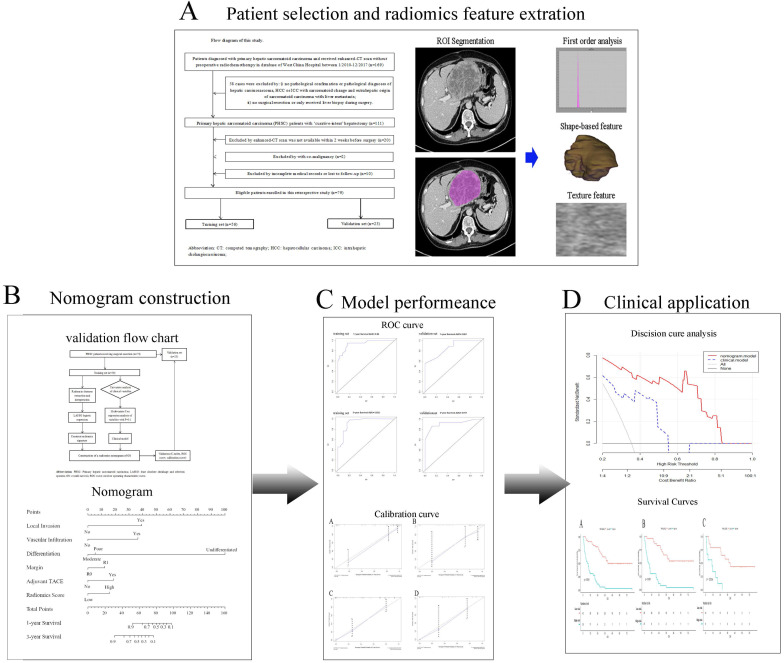
Study workflow. A Patients selection and Radiomics features extraction; B Construction of nomogram; C Comparison of model performance; D Clinical decision analysis and survival comparison. CT: computed tomographic; HCC: hepatocellular carcinoma; ICC: introhepatic cholangiocarcinoma; ROI: region of interest; TACE: transcatheter arterial chemoembolization.

**Figure 2:**
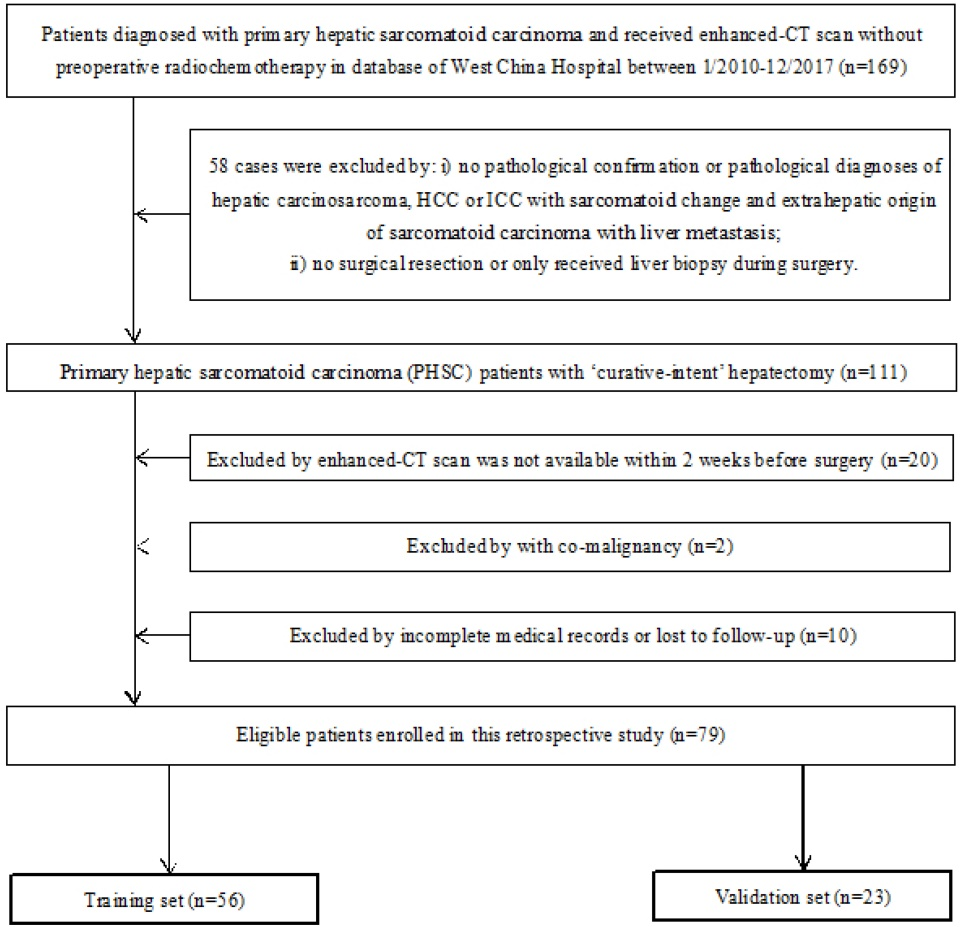
Flow diagram of patient selection of this study. CT: computed tomographic; HCC: hepatocellular carcinoma; ICC: introhepatic cholangiocarcinoma.

The inclusion criteria were: i) Patients had definite pathological diagnosis of PHSC and received curative-intent surgery; ii) patients had complete medical records and follow-up; iii) patient had available abdominal enhanced CT scan data before curative surgery. The exclusion criteria were: i) patients had definite pathological diagnosis of hepatic carcinosarcoma (CS), hepatocellular carcinoma (HCC) or intrahepatic cholangiocarcinoma (ICC) with sarcomatoid change or extrahepatic origin of sarcomatoid carcinoma with liver metastasis; ii) patients received any kind of radio chemotherapy (including transarterial chemoembolization) prior to CT scan or only received tumor biopsy during the surgery; iii) patients were diagnosed with other organ's malignancy (co-existing malignancy). The overall survival (OS) time was calculated between the surgery date and the date of death or last follow-up. The workflow of this study was shown in Figure 1 and the patient selection flow diagram was shown in Figure 2.

### CT image acquisition

All selected patients underwent enhanced abdominal CT examinations. All the instruments for CT examination were 64-detector row scanner (Brilliance 64, Philips Medical Systems, Eindhoven, the Netherlands). CT scanning was performed with 30 to 35 seconds for arterial-phase and 60 to 70 seconds for portal venous phase. Other specific CT scan parameters were reported in previous studies 26.

### Radiomics features extraction and radiomics score model building

As primary hepatic sarcomatoid carcinoma was mainly presented as a hypovascular liver mass with low intensity and more clearly defined in the venous phase. The venous phase CT imaging data was retrieved and loaded into the Local Image Features Extraction (LIFEx) software (v3.74, CEA-SHFJ, Orsay, France) to quantify pathological lesion segmentation and automated quality features 27. The ROIs were depicted freehand within the tumor lesion in venous phase images of enhanced CT by two independent radiologists. For large mass and multiple mass, ROIs were depicted within all the tumor lesions.

All enrolled patients were randomly divided into training set and validation set, with a ratio of 7:3. The Least Absolute Shrinkage and Selection Operator (LASSO) Cox regression method was used for selecting the appropriate radiomics features (features with non-zero coefficients) in the training set. The radiomics scoring model was obtained by linear combination of selected features based on non-zero coefficients of themselves. By means of the largest Youden index 28, we transformed the calculated radiomics scores from continuous variables into a categorical variable, and divide them into two categories: high score and low score.

### Selection of clinical factors and establishment of clinical model

Patients' baseline characteristics, surgical information as well as pathological information were respectfully reviewed from the hospital record system. Univariate Cox analysis and step-wise multivariate Cox regression analysis were all used to identify independent prognostic risk factors from these clinical variables. The clinical model was established by selected independent prognostic risk factors with their coefficients.

### Development and validation of nomogram

The construction of nomogram was based on the established radiomics scoring model and the independent clinical risk factors obtained from multivariate Cox analysis. The performance of nomogram was validated by calibration curve, concordance index (C-index), receiver operating characteristic (ROC) curve, decision curve analysis (DCA) 29, 30.

### Statistical analysis

All clinical variables were displayed as mean± standard deviation (continuous variable) and frequency with percentage (categorical variable). We used the Chi-square test and Student's t test to find out the difference between training and validation set. The survival curve was described by Kaplan-Meier method. In univariate Cox analysis, variables with p value <0.1 were further selected for multivariate Cox regression analysis, and p <0.05 was considered statistically significant 31. An estimated 15 patients (the real cohort of high risk and low risk patients were 36 and 43, respectively) would be needed to provide 90% power for 3-year overall survival log-rank test with a two-sided α of 0.05 (Supplement Table 2).All statistical analyses were conducted by SPSS, version 20.0 (IBM Corporation, Armonk, NY, USA) and R statistical software, version 4.0.0 (The R Foundation). The packages used in R software are glmnet, cmprsk, rms, survival, rmda and devtools.

## Results

### Patient characteristics

Finally, a total of 79 eligible patients with pathological confirmation of PHSC were included in the study. The number of men and women was 43 and 36, respectively. The mean age of all patients was 51.1 years old. The median follow-up time was 30.1 ±4.6 (95%CI: 21.1-38.9) months. The 1-year and 3-year overall survival rates were 63.3% and 35.4%, respectively. The median survival time was 23.4 months of the whole set. The 1-year and 3-year overall survival rates in training set were 64.3% and 35.7%, respectively, contrast to those of 60.9% and 34.8% in validation set, respectively. The median survival time in training and validation set were 22.9 months and 24.5 months, respectively. And no significant difference of baseline characteristics was observed between training and validation set. All patients' baseline characteristics were summarized in Table 1.

### Clinical independent preoperative prognostic factors

We included a total of 28 clinical variables in univariate Cox analysis and multivariate Cox analysis. Univariate analysis revealed 9 potential predictors: CA19-9 level, AJCC TNM staging, Child-Pugh classification, number of tumors, differentiation, local invasion, vascular infiltration, surgical margin and adjuvant transcatheter arterial chemoembolization (TACE). The p-values of these variables were all less than 0.1. We put these nine variables into the multivariate Cox regression model, and finally got five variables with p-value <0.05. These variables were considered as independent risk prognostic factors regarding to OS for patients with PHSC after surgical resection. Table 2 showed the details of univariate and multivariate analysis results.

### Feature selection and construction of radiomics score

Among the 49 radiomics features, 3 non-zero coefficient features related to prognosis were selected by LASSO regression model from training set. We combined these features linearly according to their non-zero coefficients, and finally calculated the radiomics scores of each patient. The formulas of these 3 features and their non-zero coefficients were shown in Supplement Table 1.

### Construction and validation of the nomogram

**Figure 3:**
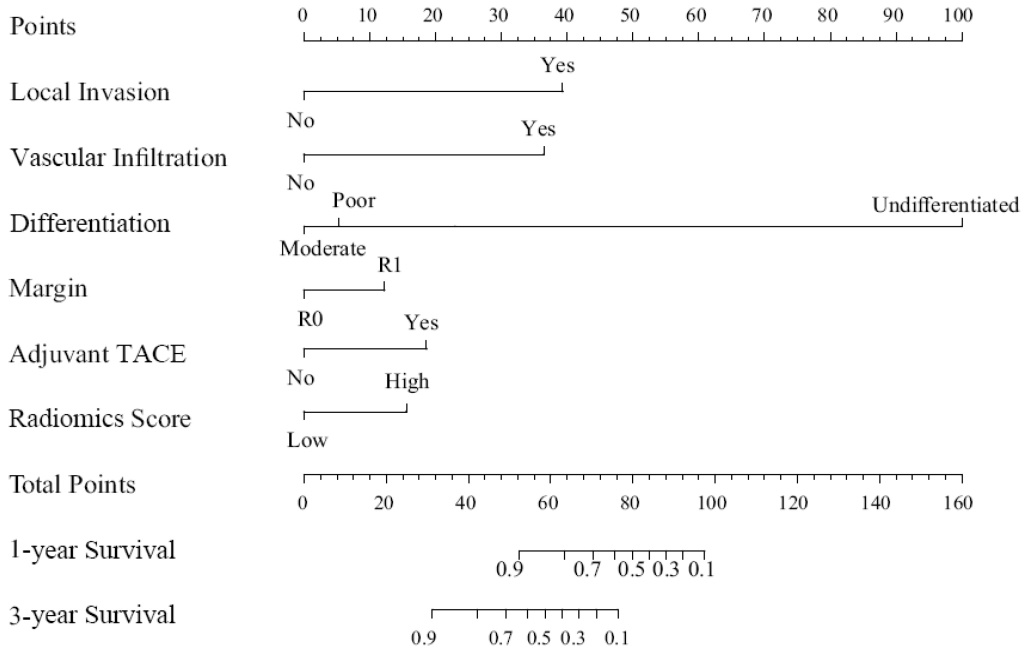
Nomogram for 1 and 3 year OS in patients with PHSC after surgical resection. OS: overall survival; PHSC: primary hepatic sarcomatoid carcinoma; TACE: transcatheter arterial chemoembolization.

**Figure 4:**
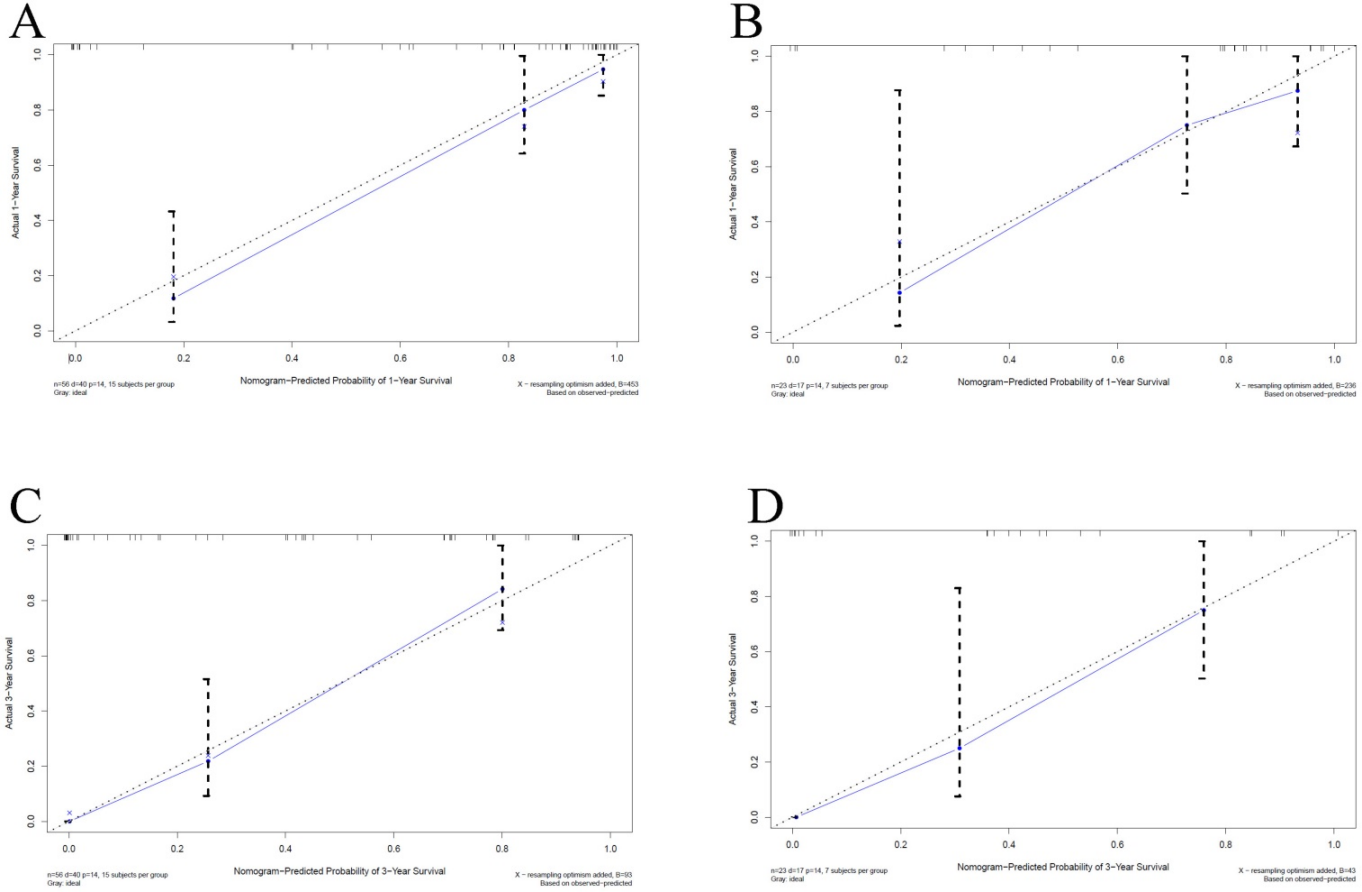
Calibration curves for overall survival (OS) at 1-year and 3-year in patients with PHSC after surgical resection. A: 1-yearsurvival rate in training set; B: 1-yearsurvival rate in validation set; C: 3-yearsurvival rate in training set; D: 3-yearsurvival rate in validation set. The horizontal axis was the survival rate predicted by the nomogram, and the vertical axis was the actual survival rate. The dashed line indicates the predicting survival rate completely fits the actual survival rate. In the training set and validation set, the prediction results of the nomogram were close to the actual results of 1-year and 3-year OS, showed the calibration curve was in good agreement. PHSC: primary hepatic sarcomatoid carcinoma.

We built a comprehensive radiomics model with five previously estimated clinical variables and radiomics score, and integrated them into a nomogram. This nomogram was constructed to predict each patient's probability of survival at 1 and 3 years after surgical resection (Figure 3). We applied C-index and calibration curve (Figure 4) to determine the predictive accuracy and discriminative ability of the nomogram. The C-index was 0.855 (95% CI, 0.798-0.912) in training set and 0.829 (95% CI, 0.702-0.956) in validation set. For 1-year OS, the area under curve (AUC) was 0.940 in training set and 0.861 in validation set. For 3-year OS, AUC of training set and validation set were 0.932 and 0.917, respectively.

### Prognosis comparison and risk stratification

**Figure 5:**
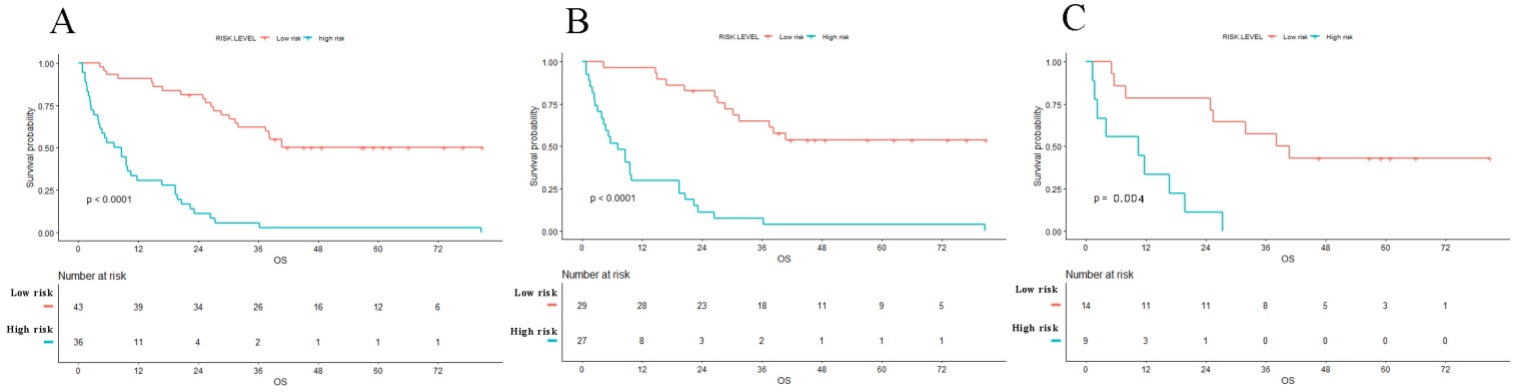
Kaplan-Meier survival curves of OS in patients with PHSC after surgical resection in entire (A) set, training set (B) and validation set (C). Patients with low risk were associated with better survival (p<0.001). OS: overall survival; PHSC: primary hepatic sarcomatoid carcinoma.

We added the scores of each nomogram variable to get the total risk score of each patient. All patients were then divided into high-risk group and low-risk group according to the cut-off value. And there were 36 high risk patients and 43 low risk patients. The result of Kaplan-Meier curve showed significantly survival difference between high-risk and low-risk patients stratified by the radiomics nomogram in entire cohort (1-year and 3-year survival rate, high risk versus low risk, 30.6% versus 90.1% and 5.6% versus 62.4%, respectively, P<0.001). The same results were also found in training set and validation set (Figure 5). The hazard ratios of nomogram, radiomics score and clinical models are shown in Table 3. This nomogram obtained the highest HR value than clinical model and radiomics score. An example of using radiomics nomogram to predict survival probability of a 35 years old male patient after surgical resection was showed in Supplement Figure 1.

### Decision curve analysis in radiomics nomogram and clinical model

**Figure 6:**
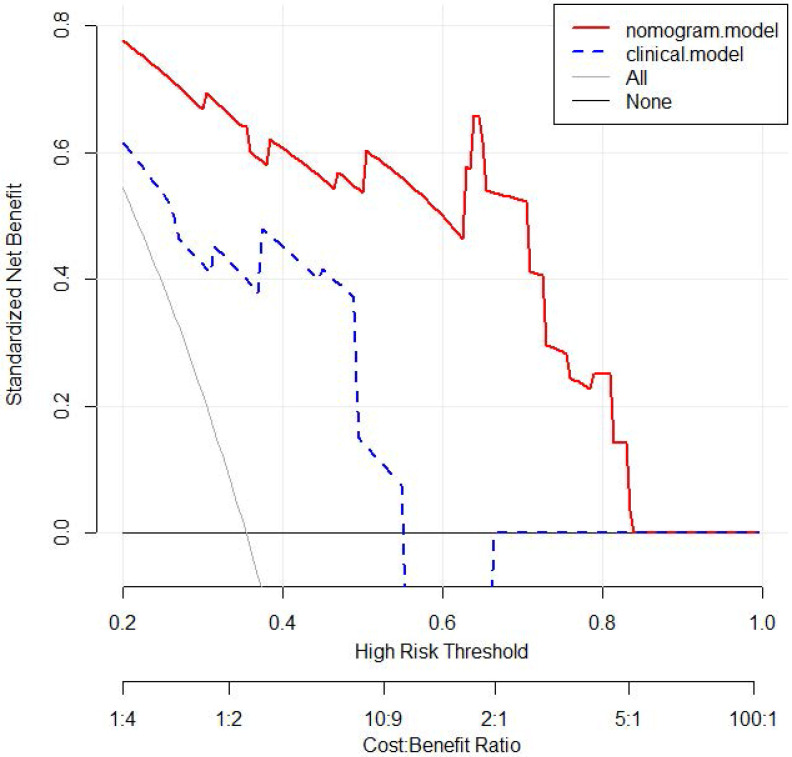
Decision curve analysis for 3-year OS of nomogram and clinical model in the validation set. The y-axis measures the net benefit. The red line represents the nomogram. The blue dotted line represents the clinical model. The gray dotted line represents the assumption that all patients dead. The black dotted line represents the assumption that no patients dead. OS: overall survival.

The nomogram showed a better performance than clinical model for almost threshold probabilities. Especially when the threshold value is less than 0.8, the net benefit of nomogram was significantly higher than that of clinical model. When the threshold value increased, the net benefits of two models were closer. Decision curve analysis results for the nomogram and clinical model were presented in Figure 6.

## Discussion

PHSC is a sophisticated type of liver malignancy with poor prognosis 1, 2, 8, 32, 33. Due to its poor prognosis, it is very important to predict the overall survival for PHSC patients. In this study, we found that positive surgical margin, vascular infiltration, local invasion, differentiation, adjuvant TACE and image score were all independent factors relating to prognosis through univariate and multivariate analysis. We developed a comprehensive nomogram based on these independent risk factors, and then stratified patients into high risk and low risk set. There was significantly different survival time between high risk and low risk patients (average survival time, high risk versus low risk, 12.1 months versus 52.7 months, P<0.001). To our knowledge, this was the first comprehensive CT-based radiomics nomogram for predicting overall survival of PHSC after curative-intent surgery.

Curative surgery can obviously prolong the overall survival of PHSC, and radical resection can obtain significant higher survival time than palliative resection. The median survival time was reported to 15.6-20.5 months for patients who received radical resection contrast to 7.6-7.9 months for those underwent palliative resection 1, 2. In the present study, the median survival time for R0 resection was 25.5 months contrast to 10.2 months of R1 resection (p=0.017). In further, previous studies reported vascular infiltration and local invasion were occurred more often in PHSC than conventional hepatocellular carcinoma and were associated with worse prognosis 1, 2, 13, 34, 35. In the present study, vascular infiltration and local invasion were found in 21.5% and 35.4% of all patients, respectively, which were similar to the reported incidence of 28.6% and 21.4%, respectively 2. Besides, high incidence of vascular infiltration and local invasion could result in significantly worse prognosis, with reported median disease-free survival time of 3.2 months and 3.6 months in patients with vascular infiltration and local invasion, respectively. In this study, we found that patients with vascular infiltration suffered significantly lower survival time (median survival time, vascular infiltration versus no vascular infiltration, 11.7 months versus 25.5 months, P=0.044) than those with no vascular infiltration. Besides, the median survival time of patients with local invasion were significantly lower than patients without local invasion (local invasion versus no local invasion, 32.0 months versus 12.6 months, P<0.001).

Tumor differentiation was associated with prognosis of liver tumor; high grade of differentiation can lead to poor prognosis. Previous study revealed that the sarcomatoid hepatocellular carcinoma was associated with more advanced histopathological grade than conventional hepatocellular carcinoma 36. Moreover, a population-based study also found sarcomatoid hepatocellular carcinoma were relating to worse tumor grade than conventional HCC through propensity score matching 37. In this study, patients with advanced differentiation grade (poor differentiation and undifferentiation) were about 49.4% of all patients, which was similar to the previous reported incidence of 52.9% 37. In addition, we found that advanced differentiation grade was associated with worse prognosis than moderate differentiation (average survival time, advanced grade versus moderate grade, 34.0 months versus 55.6 months, P=0.019). The survival curves stratified by surgical margin, vascular invasion, local invasion, and differentiation grade were shown in Supplement Figure 2.

Radiomics is a renewed objective and quantitative evaluation method of cancer heterogeneity, which was better than sampling biopsy which could assess only a small part of the tumor 38, 39. Many previous studies had confirmed the flexibility and effectiveness of CT radiomics in recurrence and prognostic prediction of liver malignancies 40-44. Moreover, the nomogram based on radiomics may obtain higher prediction value than clinical model only 23-25. In the present study, we integrated three radiomics features which were extracted from 49 radiomics features and five clinical independent risk factors into a comprehensive radiomics nomogram for prognostic prediction. The DCA showed that the net benefit was higher in radiomics nomogram than clinical model when the threshold less than 0.8. Besides, when compared the model performance with three models, we also found that the HR in nomogram was higher than that either in radiomics score or clinical model. Our results were in line with the previous studies.

Surprisingly, the present study found that patient cannot obtain survival benefit in adjuvant TACE. Previous studies found that PHSC was mainly present as a hypovascular liver mass with low intensity 5, 8, 45, 46, albeit another study insisted that PHSC may serve as a hypervascular mass 2. The nature of hypovascularity implied that TACE might have feeble survival benefit on these patients. In the contrary, patient who received TACE often associated with advanced tumor stage. In the present study, we found that adjuvant TACE was a hazard factor associated with worse prognosis (average survival time, TACE versus no TACE, 11.0 months versus 40.2 months, P<0.01). The result was agreed with the previous study of Wang et.al which reported the average survival time in TACE +operation and operation without TACE was 10.6 months and18.3 months, respectively 1. The possible reason might be hypovascular nature of tumor and the lack of appropriate selection standard for TACE in PHSC patients. Thus, further studies focus on identifying appropriate patients for additional adjuvant TACE for PHSC after surgical resection could be conducted to verify the results.

This study still has some limitations. First, the nature of retrospective study may generate selective and withdraw bias. Second, this study was absence of external validation. Thus, future prospective and multicenter studies should be conducted to validate the results. Third, although PHSC was considered as a single tumor mixed with carcinoma and sarcomatoid components, the proportion of each component may be various in each patient. However, it was very difficult to clearly evaluate the proportion of each component in current immunohistochemistry. Thus, further radiomics studies referred to the features of high-quality histopathological images should be conducted to reduce this bias microcosmically.

## Conclusion

PHSC often presented with poor prognosis. The radiomics nomogram established in this study may be useful in predicting the overall survival rate of PHSC patients after curative-intent surgery and help to identify high risk patients who may obtain feeble survival benefit from surgical resection.

## Key Points

- The radiomics nomogram showed good performance for prediction of overall survival in primary hepatic sarcomatoid carcinoma after surgical resection.
- The prognosis of high-risk and low-risk patients was significantly different.
- The radiomics nomogram could benefit to make decision in identifying high risk patients.

## Supplementary Material

Supplementary figures and tables.

## Figures and Tables

**Table 1:** Characteristics of patients with PHSC after surgical resection

Characteristic	PHSC patients			*P*
	Entire set (n=79)	Training set (n=56)	Validation set (n=23)	
Sex, n (%), male	43 (54.4)	28 (50)	15 (65.2)	0.22
Age (y), mean ± SD	51.1±13.4	50.8±13.5	51.8±13.4	0.765
ALT (U/L), mean ± SD	54.5±44.6	55.2±49.2	49.3±31.3	0.53
AST (U/L),mean ± SD	52.0±35.8	50.6±36.7	53.4±33.8	0.58
Serum albumin (g/L), mean ± SD	41.3±4.8	41.5±4.9	40.9±4.4	0.61
TBIL (umol/L), mean ± SD	15.2±6.3	15.7±6.7	14.1±5.2	0.26
Prothrombin time (s), mean ± SD	12.5±1.9	12.4±1.9	12.7±1.7	0.48
AFP (ng/ml), mean ± SD	194.7±333.7	239.6±378.1	85.3±141.2	0.06
CA19-9 (U/ml), mean ± SD	45.4±127.8	53.0±150.4	27.0±28.9	0.22
CEA (ng/ml), mean ± SD	3.1±3.9	3.4±4.5	2.5±1.5	0.17
Child-Pugh class, n, A/B	72/7	51/5	21/2	0.97
Tumor size (cm), mean ± SD	7.1±4.0	6.6±3.9	7.1±3.2	0.52
Number of tumor, n (%)				0.79
Single	46 (58.2)	33 (58.9)	13 (56.5)	
Multiple	33 (41.8)	23 (41.1)	10 (43.5)	
Capsule formation, n (%)	8 (10.1)	7 (12.5)	1 (4.3)	0.28
Local invasion, n (%)	28 (35.4)	19 (33.9)	9 (39.1)	0.67
Vascular invasion, n (%)	17 (21.5)	13 (23.2)	4 (17.3)	0.56
Bile duct invasion, n (%)	10 (12.7)	7 (12.5)	3 (13.0)	0.95
Lymph node metastasis, n (%)	9 (11.4)	7 (12.5)	2 (8.6)	0.61
Cancerous component, HCC/ICC	43/36	31/25	12/11	0.80
Differentiation, n (%)				0.59
Moderately	8 (10.1)	5 (8.9)	3 (13.0)	
Poorly	35 (44.3)	27 (48.2)	8 (34.8)	
Undifferentiated	4 (5.1)	3 (5.4)	1 (4.3)	
NA	32 (40.5)	21 (37.5)	11 (47.8)	
8th AJCC staging, n (%)				0.72
I+II	40(50.6)	28 (50.0)	12 (52.2)	
IIIA+IIIB	30 (38.0)	21 (37.5)	9 (39.1)	
IVA	9 (11.4)	7 (12.5)	2 (8.7)	
Extent of hepatectomy, n (%)				0.17
Minor liver resection	45 (57.0)	35 (62.5)	10 (43.5)	
Major liver resection	31 (39.2)	19 (33.9)	12 (52.2)	
Liver transplantation	3 (3.8)	2 (3.6)	1 (4.3)	
Anatomy resection, n (%), yes	16 (20.3)	11 (19.6)	5 (21.7)	0.84
Transfusion, n (%), yes	14 (17.7)	10 (17.8)	4 (17.4)	0.96
Blood loss (ml), mean ± SD	660.8±778.3	626.8±651.4	743.5±1037.7	0.62
Margin, n (%)				0.95
R0	69 (87.3)	49 (87.5)	20 (87.0)	
R1	10 (12.7)	7 (12.9)	3 (13.0)	
Adjuvant TACE, n (%)				0.98
Yes	17 (21.5)	12 (21.4)	5 (21.7)	
No	62 (78.5)	44 (78.3)	18 (78.3)	
Hospital stay (d), mean ± SD	13.5±7.6	13.2±7.6	14.3±7.5	0.59
Total hospitalization expenses (USD), mean ± SD	7913.0±8367.5	7850.8±8185.8	8064.5±8982.2	0.92

Abbreviation: PHSC: primary hepatic sarcomatoid carcinoma; SD: standard deviation; ALT: alanine aminotransferase; AST: aspartate aminotransferase; TBIL: total bilirubin; AFP: alpha-fetoprotein; CEA: carcinoembryonic antigen; HCC: hepatocellular carcinoma; ICC: introhepatic cholangiocarcinoma; NA: not applicable; AJCC: American joint Committee on Cancer; TACE: transcatheter arterial chemoembolization; USD: United States dollar.

**Table 2:** Univariate analysis and multivariate Cox regression analysis of clinical factors in the training cohort

Variable	Univariate analysis (*p* value)	Multivariate analysis HR (95%CI)	*p* value
Sex	0.781		
Age, years	0.106		
Hepatitis	0.889		
ALT level, U/L	0.896		
AST level, U/L	0.787		
ALB level, g/L	0.225		
TBIL level, umol/L	0.806		
AFP level, ng/m	0.779		
PT, s	0.685		
CA19-9 level, U/ml	0.059	1.001 (1.000-1.003)	0.067
CEA level, ng/ml	0.220		
Tumor size, cm	0.353		
8th AJCC staging	0.001		
I+II		Ref	
III		Ref	
IVA		1.651 (0.633-4.309)	0.305
Child-Pugh classification	0.011		
A		Ref	
B		1.221 (0.480-3.106)	0.676
Number of tumors	0.001		
Single		Ref	
Multiple (>1)		0.769 (0.370-1.599)	0.270
Differentiation	0.003		
moderate		Ref	
Poor		Ref	
Undifferentiated		1.597 (1.143-2.237)	0.006
Capsule formation	0.232		
Local invasion	0.001		
No		Ref	
Yes		3.159 (1.675-5.957)	<0.001
Vascular infiltration	0.047		
No		Ref	
Yes		4.417 (2.112-9.236)	<0.001
Bile duct invasion	0.816		
Lymph node metastasis	0.012		
Cancerous component,HCC/ICC	0.503		
Anatomy resection	0.401		
Extent of hepatectomy	0.281		
Intraoperative blood transfusion	0.366		
Blood loss, ml	0.674		
Surgical margin	0.034		
R0		Ref	
R1		3.232 (1.486-7.027)	0.003
Adjuvant TACE	0.001		
No		Ref	
Yes		2.327 (1.115-4.858)	0.025

Abbreviation: ALT: alanine aminotransferase; AST: aspartate aminotransferase; TBIL: total bilirubin; AFP: alpha-fetoprotein; PT: prothrombin time; CEA: carcinoembryonic antigen; NA: not applicable; AJCC: American joint Committee on Cancer; HCC: hepatocellular carcinoma; ICC: introhepatic cholangiocarcinoma; TACE: transcatheter arterial chemoembolization; ref: reference.

**Table 3:** Comparison of hazard ratio of three models

Model	HR (95%CI)	*p*-value
**Radiomics score**		0.009
Low risk	Ref	
High risk	2.047 (1.198-3.498)	
**Clinical model**		<0.001
Low risk	Ref	
High risk	5.205 (2.644-10.648)	
**Nomogram**		<0.001
Low risk	Ref	
High risk	7.111 (3.933-12.858)	

Abbreviation: HR: hazard ratio, Ref: reference.

## Abbreviations

PHSC: primary hepatic sarcomatoid carcinoma
DCA: decision curve analysis
LASSO: least absolute shrinkage and selection operator
HR: hazard ratio
SD: standard deviation
ALT: alanine aminotransferase
AST: aspartate aminotransferase
TBIL: total bilirubin
AFP: alpha-fetoprotein
CEA: carcinoembryonic antigen
SHC: sarcomatoid hepatocellular carcinoma
ISCC: intrahepatic sarcomatoid cholangiocarcinoma
WHO: World Health Organization
CT: computed tomographic
HCC: hepatocellular carcinoma
ICC: introhepatic cholangiocarcinoma
CS: carcinosarcoma
ROC: receiver operating characteristic
NA: not applicable
OS: overall survival
AJCC: American joint Committee on Cancer
TACE: transcatheter arterial chemoembolization
AUC: area under curve
USD: United States dollar
