# Supplementary material for: Development and validation of a comprehensive radiomics nomogram for prognostic prediction of primary hepatic sarcomatoid carcinoma after surgical resection
Source: Int J Med Sci. 2021 Feb 6;18(7):1711–20. doi: 10.7150/ijms.53602 (PMC7976557; doi:10.7150/ijms.53602)
Supplement: Supplementary file 1 — Supplementary figures and tables. [file ijmsv18p1711s1.pdf]

Supplement figure 1. An example of using radiomics nomogram to predict survival probability of a 35 years old male patient after surgical resection. ROI: region of interest; TACE: transcatheter arterial chemoembolization.

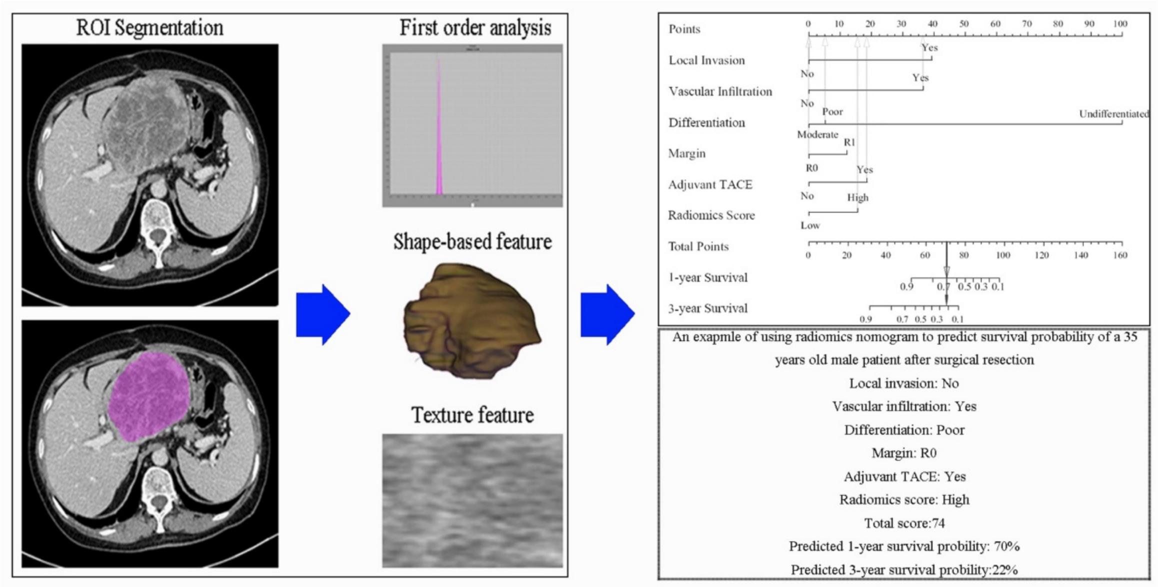

Supplement figure 2. The survival curves stratified by surgical margin, vascular invasion, local invasion, and differentiation grade. A: survival curves stratified by surgical margin; B: survival curves stratified by vascular invasion; C: survival curves stratified by local invasion; D: survival curves stratified by differentiation grade.

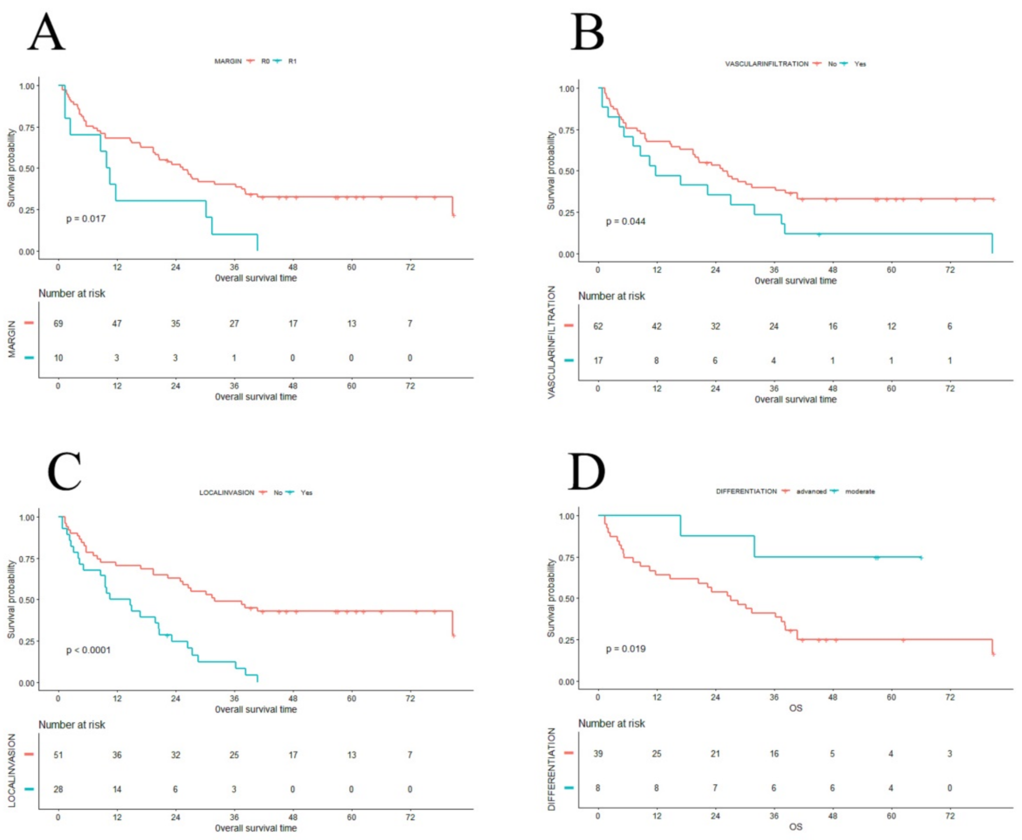

Supplement table 1. The formulas for constructing the radiomics scoring models.

Model	Formula
radiomics score	-0.0001249365SHAPE_Sphericity+3.2096802376GLRLM_LGRE+2.5467404092GLRLM_SRLGE

Supplement table 2. Power analysis of the study.

**High risk group/ Low risk group**	value	**High risk group/ Low risk group**	value
Two-sided α	0.05	Two-sided α	0.05
1-year overall survival rate (**High risk group**)	0.306	3-year overall survival rate (**High risk group**)	0.056
1-year overall survival rate (**Low risk group**)	0.901	3-year overall survival rate (**Low risk group**)	0.624
Assuming hazard ratio	7.01	Assuming hazard ratio	2.5
Power	90%	Power	90%
Estimated patients number (**High risk group**)	3	Estimated patients number (**High risk group**)	7
Estimated patients number (**Low risk group**)	4	Estimated patients number (**Low risk group**)	8
